# Aminoacyl-tRNA synthetases

**DOI:** 10.1261/rna.071720.119

**Published:** 2020-08

**Authors:** Miguel Angel Rubio Gomez, Michael Ibba

**Affiliations:** Center for RNA Biology, The Ohio State University, Columbus, Ohio 43210, USA Department of Microbiology, The Ohio State University, Columbus, Ohio 43210, USA

**Keywords:** aminoacyl-tRNA synthetases, tRNA, protein translation

## Abstract

The aminoacyl-tRNA synthetases are an essential and universally distributed family of enzymes that plays a critical role in protein synthesis, pairing tRNAs with their cognate amino acids for decoding mRNAs according to the genetic code. Synthetases help to ensure accurate translation of the genetic code by using both highly accurate cognate substrate recognition and stringent proofreading of noncognate products. While alterations in the quality control mechanisms of synthetases are generally detrimental to cellular viability, recent studies suggest that in some instances such changes facilitate adaption to stress conditions. Beyond their central role in translation, synthetases are also emerging as key players in an increasing number of other cellular processes, with far-reaching consequences in health and disease. The biochemical versatility of the synthetases has also proven pivotal in efforts to expand the genetic code, further emphasizing the wide-ranging roles of the aminoacyl-tRNA synthetase family in synthetic and natural biology.

## THE AMINOACYL-tRNA SYNTHETASES

Aminoacyl-tRNA synthetases (aaRSs) are universally distributed enzymes that catalyze the esterification of a tRNA to its cognate amino acid (i.e., the amino acid corresponding to the anticodon triplet of the tRNA according to the genetic code) (Ibba and Soll 2000; Pang et al. 2014). The product of this reaction, an aminoacyl-tRNA (aa-tRNA), is delivered by elongation factors to the ribosome to take part in protein synthesis. The discovery of the aaRSs and their role in protein synthesis began in the 50s and 60s when it was reported that amino acids were required to undergo an activation process in order to take part in protein synthesis (Hoagland 1955; Zamecnik et al. 1958). The discovery of tRNA (Hoagland et al. 1958), the bridging molecule foretold by Crick in his adaptor hypothesis, led to the identification of the enzymes responsible for establishing the link between the nucleotide and amino acid world, the aminoacyl-tRNA synthetases (Hoagland et al. 1958). AaRSs fulfill two extremely important roles in translation: not only do they provide the building blocks for protein synthesis, they are also the only enzymes capable of implementing the genetic code (Woese et al. 2000; Banik and Nandi 2012). Aminoacyl-tRNA synthetases are named after the aminoacyl-tRNA product generated, as such, methionyl-tRNA synthetase (abbreviated as MetRS) charges tRNA^Met^ with methionine. In eukaryotes, an alternative nomenclature is often used using the one-letter code of the amino acid (MARS) and a number is added to refer to the cytosolic (MARS1) or the mitochondrial (MARS2) variants. A total of 23 aaRSs have been described so far, one for each of the 20 proteinogenic amino acids (except for lysine, for which there are two) plus pyrrolysyl-tRNA synthetase (PylRS) and phosphoseryl-tRNA synthetase (SepRS), enzymes with a more restricted distribution that are only found in some bacterial and archaeal genomes (Cusack et al. 1990; Cusack 1995; Arnez and Moras 1997; Mukai et al. 2017a). It is also worth noting that in eukaryotes the protein synthesis machineries of mitochondria and chloroplasts generally utilize their own, bacterial-like sets of synthetases and tRNAs that are distinct from their cytosolic counterparts (Tzagoloff et al. 1990; Bonnefond et al. 2007).

**FIGURE 1.**
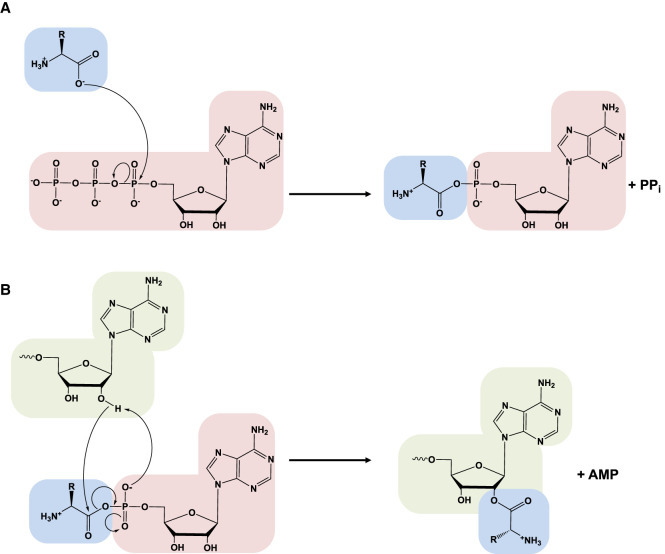
The aminoacylation reaction. In the first step (*A*), the amino acid (blue) is activated with ATP (red) in the synthetase active site (not depicted), forming the aminoacyl-AMP and releasing PP_i_. (*B*) The amino acid is transferred to the tRNA (green) and AMP is released (depicted in the image transfer to the 2′-OH characteristic of class I aaRS, while in class II transfer happens with a 3′-OH).

The aminoacyl-tRNA synthetases catalyze a two-step reaction that leads to the esterification of an amino acid to the 3′ end of a tRNA along with the hydrolysis of one molecule of ATP, yielding aminoacyl-tRNA, AMP, and PP_i_. In the first step, termed amino acid activation, both the amino acid and ATP bind to the catalytic site of the enzyme, triggering a nucleophilic attack of the α-carboxylate oxygen of the amino acid to the α-phosphate group of the ATP, condensing into aminoacyl-adenylate (aa-AMP), which remains bound to the enzyme, and PP_i_, which is expelled from the active site (Fig. 1A). Although tRNA is usually not required for this first step, certain synthetases (GlnRS, GluRS, ArgRS, and class I LysRS) (Ravel et al. 1965; Mitra and Mehler 1967; Ibba et al. 2001) do require the tRNA species for productive amino acid activation. In the second part of the reaction, the hydroxyl group of the adenine 76 nt attacks the carbonyl carbon of the adenylate, forming aminoacyl-tRNA and AMP (Fig. 1B). While the two-step aminoacylation reaction is universally conserved, the aaRSs that catalyze it show extensive structural, and in some instances functional, diversity as described in detail below.

### Classification of aminoacyl-tRNA synthetases

The 23 known aaRSs can be divided into two major classes based on the architecture of their active sites (Cusack et al. 1990; Eriani et al. 1990a; Burbaum and Schimmel 1991; Cusack 1997; Ribas de Pouplana and Schimmel 2001b; O'Donoghue and Luthey-Schulten 2003). In class I synthetases, the catalytic domain bears a dinucleotide or Rossman fold (RF) featuring a five-stranded parallel β-sheet connected by α-helices and is usually located at or near the amino terminus of the protein. This RF contains the highly conserved motifs HIGH and KMSKS (Brick et al. 1989; Rould et al. 1989; Schmidt and Schimmel 1994), separated by a connecting domain termed connective peptide 1 (CP1) (Starzyk et al. 1987). Class II active site architecture is organized as seven-stranded β-sheets flanked by α-helices and features three motifs which show a lesser degree of conservation than those in class I (Cusack et al. 1990; Eriani et al. 1990a; Arnez et al. 1995). Both classes also exhibit pronounced differences in their modes of substrate binding. Class I aaRSs bind the minor groove of the tRNA acceptor stem (with the exceptions of TrpRS and TyrRS) and aminoacylate the 2′-OH of the ribose of A76, while class II approach tRNA from the major groove (Ruff et al. 1991) and transfer amino acid to the 3′-OH (Sprinzl and Cramer 1975) (with the exception of PheRS) (Sprinzl and Cramer 1975; Ruff et al. 1991; Ibba et al. 2001). The mode of ATP binding is also different between both classes, being bound in an extended configuration in class I (Brick and Blow 1987; Brick et al. 1989; Rould et al. 1989), while class II binds a bent configuration with the γ-phosphate folding back over the adenine ring (Perona and Hadd 2012). The kinetics of the aminoacylation reaction can also be used as a distinctive mechanistic signature, as aminoacyl-tRNA release is the rate limiting step for class I enzymes (except for IleRS and some GluRS) while for class II it is the amino acid activation rate instead (Fersht 1977; Perona et al. 1991; Kaminska et al. 2001).

Class I and II can be further divided into different subgroups based upon phylogenetic analysis, comparison of structural and mechanical characteristics and domain organization (Table 1). Although there is consensus in the division of class II synthetases into three subgroups (a, b, and c), the classification of class I is more complex, with some authors classifying them into three subgroups (Cusack 1995; Ribas de Pouplana and Schimmel 2001b; First 2005) while others propose up to five subclasses (Perona and Hadd 2012; Valencia-Sánchez et al. 2016). Interesting relations between the aaRSs and their amino acid substrates emerge when considering the grouping into subclasses. For example, subclass Ia recognizes aliphatic amino acids such as Leu, Ile, and Val and thiolated amino acids such as Met and Cys, while class Ic aaRSs activate the aromatic amino acids Tyr and Trp. Interestingly, similar correlations exist within the class II enzymes. For example, class Ib enzymes activate charged amino acids such as Lys, Glu, and Gln, while their class IIb counterparts activate Lys, Asp, and Asn, also polar amino acids.

**TABLE 1.**
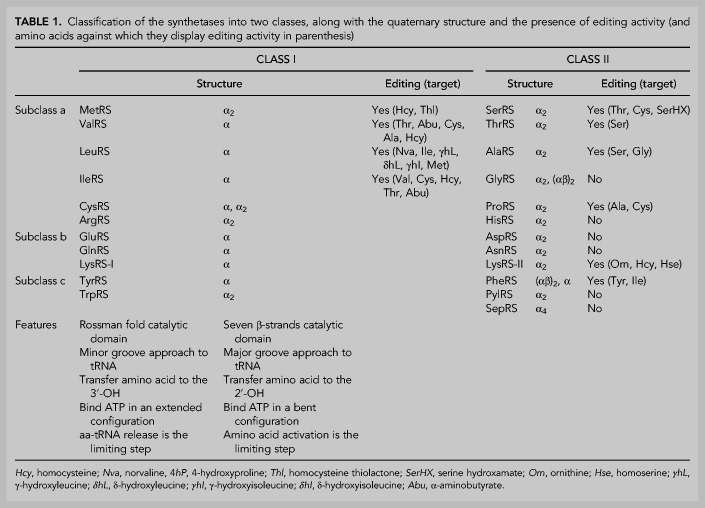
Classification of the synthetases into two classes, along with the quaternary structure and the presence of editing activity (and amino acids against which they display editing activity in parenthesis)

The structural diversification of the aaRSs can be correlated both with the recognition of structurally and chemically diverse cognate substrates, and with the need to exclude near- and noncognate amino acids. To prevent the use of mischarged tRNAs in protein synthesis, some synthetases have evolved editing activities that specifically target and hydrolyze misactivaed amino acids and/or misacylated tRNAs. The editing activity may reside in the catalytic site, in separate domains or even in freestanding separate proteins. In class I synthetases, the editing activity is usually located in the connecting peptide CP1 while in class II this activity can be located in different domains (Schmidt and Schimmel 1994, 1995; Lin and Schimmel 1996; Giege et al. 1998; Nureki et al. 1998; Dock-Bregeon et al. 2000). The editing domains and mechanism used will be discussed in detail below.

### Origin and evolution of aaRSs

AaRSs are believed to have originated very early in evolution and it is thought that an almost complete set was already present within the last universal common ancestor (LUCA) (Nagel and Doolittle 1995; Woese et al. 2000; Fournier et al. 2011). The aminoacyl-tRNA synthetases are a unique family of proteinaceous enzymes, as they are the only proteins that are able to decode the rules of the genetic code, all while being translated following those same rules. This apparent dilemma has made unveiling the evolutionary origin of synthetases particularly intriguing. As mentioned above, both classes of synthetases approach the tRNA from different sides and it is possible to simultaneously model the docking of pairs of enzymes of each class to a single tRNA without major steric hindrances (Ribas de Pouplana and Schimmel 2001b). This complementary recognition of the major and minor grooves of the tRNA acceptor stem is the basis of a proposed evolutionary model in which both ancestors of each class arose form a single gene. Under this scenario, usually known as the Rodin–Ohno hypothesis, the gene of the ancestral aminoacyl-tRNA syntethase could be read bidirectionally, and each of the opposite strands would code for the ancestor of class I and class II, respectively. Both would be able to interact with the tRNA molecule and charge it with different amino acids. Although only an extremely simplified genetic code could be sustained with these two enzymes, subsequent events of gene duplication would configure the set of synthases as it is known today (Rodin and Ohno 1995; Kunst et al. 1997; Carter and Duax 2002; Rodin et al. 2009; Martinez-Rodriguez et al. 2015). As the study of the evolution of aaRSs involves exploring scenarios before the LUCA, experimental research is often challenging. One useful strategy is to compare enzyme anatomy by superposing tridimensional models aiming at unveiling the most basic functional and invariant core of the enzyme. This extremely reduced version of an aaRS has been termed an urzyme (“ur” meaning primitive, original, earliest) and usually comprises the amino acid activation and acyl-transfer active sites of the full-length enzyme. Despite containing only slightly more than a hundred amino acids, urzymes still retain the basic catalytic capabilities of the synthetase (Augustine and Francklyn 1997; Pham et al. 2010; Carter 2017; Carter and Wills 2019). For example, a 130 amino acid long tryptophanyl-tRNA synthetase urzyme has been shown to accelerate Trp activation 109-fold (compared to the spontaneous activation rate) (Pham et al. 2007) and similar results have been achieved with HisRS (Li et al. 2011). Regarding selectivity, these ancestral urzymes would operate on an extremely basic code, each class favoring hydrophobic, or hydrophilic amino acids rather than specific ones. This binary code would create the hydrophobic cores and solvent interfaces of the primordial globular proteins (Pham et al. 2007). After their original inception, subsequent duplication, specialization, and domain acquisitions events would complete the actual set of synthetases (Rodin and Ohno 1995; Augustine and Francklyn 1997; Woese et al. 2000; O'Donoghue and Luthey-Schulten 2003).

### Organisms with incomplete sets of aaRSs

**FIGURE 2.**
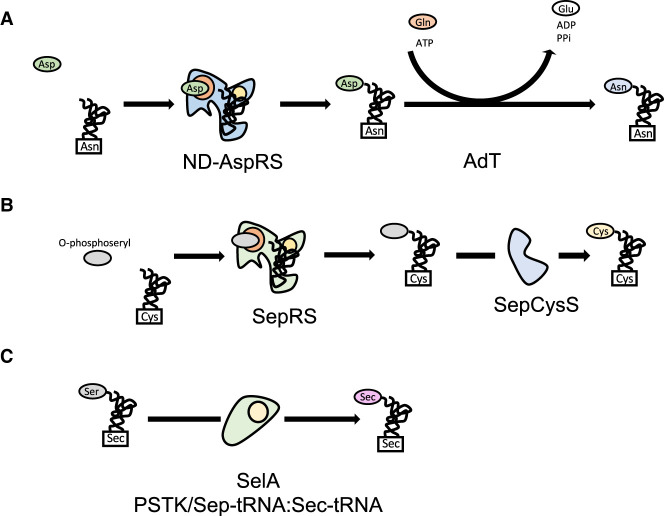
Indirect aminoacylation pathways. (*A*) Asn and Gln. tRNA^Asn^ is mysaspartylated by a ND-AspRS. The resultant Asp-tRNA^Asn^ is converted to Asn-tRNA^Asn^ by the glutamine-dependent amidotransferase (AdT). The process is similar for Gln-tRNA^Gln^. (*B*) Cysteine. tRNA^Cys^ is charged with O-phosphoserine by dedicated synthetases and further modified to cysteine by SepCysS to yield Cys-tRNA^Cys^. (*C*) Selenocysteine. SelA (Bacteria) or PSTK followed by Sep-tRNA:Sec-tRNA synthase (Archaea and Eukarya) modify a previously charged SertRNA^Sec^ from serine to selenocysteine.

As proteins are made of 20 L-amino acids, it would be expected for every organism to have a complete set of 20 aaRSs and, consistent with this, mutations in synthetase genes often lead to diseases if not lethality. Surprisingly, complete genome analysis has found numerous instances, especially among bacterial and archaeal genomes, in which ORFs encoding for synthetases are missing (Bult et al. 1996; Kunst et al. 1997; Smith et al. 1997; Cathopoulis et al. 2007). GlnRS is the most common absence in the synthetase set, being absent in archaeal genomes and often missing from many bacteria and eukaryotic organelles. Another aaRS often missing is AsnRS. These organisms accomplish the charging of tRNA^Asn^ and tRNA^Gln^ via an indirect two-step route that involves a nondiscriminating (ND) synthetase and an amidotransferase (AdT) complex (Fig. 2A). In the first step, a nondiscriminating Asp or GluRS incorrectly charges the tRNA producing Asp-tRNA^Asn^ or Glu-tRNA^Gln^ (Wilcox 1969; Lapointe et al. 1986; Schön et al. 1988; Curnow et al. 1996). The misacylated tRNA is the substrate of an amidotransferase complex that catalyzes the transamidation of the amino acid using ATP and an amino group donor from glutamine (Curnow et al. 1997; Raczniak et al. 2001). In order to prevent mistranslation of Asn or Gln codons, the product of these ND-aaRSs must not be liberated before reaching the amidotransferase. This goal is achieved by forming a complex with the adT, the synthetase and the tRNA termed the transamidosome, that channels the aminoacyl-tRNA directly to the AdT (Bailly et al. 2007; Rathnayake et al. 2017). To ensure accurate decoding and avoid relying on a ND synthetase, some bacteria use a set of duplicated enzymes. For example, *Helicobacter pylori* has a set of two GluRS: GluRS1 is a discriminating enzyme used for decoding Glu codons while GluRS2, its nondiscriminating counterpart, is used for indirect synthesis of tRNA^Gln^ (Salazar et al. 2003; Skouloubris et al. 2003). This complementarity of functions ensures an accurate decoding of the genetic message.

The AdT complex in Bacteria is made up of three proteins called GatCAB while in some mitochondria the subunit GatC is replaced by the longer GatF (Araiso et al. 2014). In archaeal genomes, the Adt is composed of a tetramer made by GatDE (Tumbula et al. 2000; Feng et al. 2004). Interestingly, phylogenetic analyses of bacterial genomes that contain GlnRS suggest a eukaryotic origin and posterior acquisition by bacterial phyla via horizontal gene transfer (Lamour et al. 1994; Brown and Doolittle 1999). In some organisms, the biosynthetic genes for Asn or Gln are missing, as in the case of *S. aureus*, that lacks genes for asparagine biosynthesis and relies on an ND-AspRS and the indirect route to synthetize this amino acid on the tRNA (Mladenova et al. 2014). It has been proposed that a bona fide GlnRS and AsnRS emerged in a eukaryotic post-LUCA environment, suggesting Asn and Gln to be late additions to the genetic code (Ribas de Pouplana and Schimmel 2001a).

Some methanogenic archaea lack the CysRS gene and use an indirect route for charging tRNA^Cys^ (Tumbula et al. 1999). This route relies on a noncanonical class II aaRS, termed *o*-phosphoseryl-tRNA synthetase (SepRS) that charges tRNA^Cys^ with the nonproteinogenic amino acid *o*-phosphoserine (Sauerwald et al. 2005). The *o*-phosphoseryl-tRNA^Cys^ intermediate is then further modified by Cys-tRNA synthase (SepCysS) into cysteine (Fig. 2B; Sauerwald et al. 2005; Mukai et al. 2017a). In some methanogenic archaea, such as *Methanocaldococcus jannaschii*, *Methanothermobacter thermautotrophicus,* and *Methanopyrus kandleri*, the *cysE* ORF encoding for one of the genes for cysteine biosynthesis is missing and the mechanism described above seems to be the only route available for cysteine biosynthesis (Ambrogelly et al. 2004; Feng et al. 2004).

### Nonhomologous duplication of aminoacyl-tRNA synthetases

#### LysRS

LysRS is the only synthetase known to date with representatives in both structural classes. Class II LysRS is the most abundant form, present in most organisms, while the class I LysRS is found mostly in archaea and some bacteria, apparently as a result of horizontal gene transfer (Eriani et al. 1990b; Ibba et al. 1997b). Although only one class of LysRS is found in most organisms, *Methanosarcinaceae* archaea and some other isolated species such as *Nitrosococcus oceani* and *Bacillus cereus* have both classes (Polycarpo et al. 2003). Structures for both forms have been resolved and shown to use similar mechanisms for substrate recognition and even recognize the same tRNA determinants (Terada et al. 2002). Phylogenetic analyses show that both enzymes have a different evolutionary origin and are usually presented as an example of convergent evolution (Ibba et al. 1997a).

#### GlyRS

Another example of duplicated synthetases that present two isoforms of different origin is GlyRS. The most common form in bacteria is a tetramer (α_2_β_2_) that is classified as IIc, while archaea, eukaryotes and some bacteria possess a dimeric form (α_2_) classified as IIa (Freist et al. 1996; O'Donoghue and Luthey-Schulten 2003; Perona and Hadd 2012). Although both forms share the characteristic active site for class II synthetases, the other structural elements of this domain are different for the two forms, the most striking difference being the amino acid recognition pocket. In the dimeric GlyRS, the amino acid is recognized by three negatively charged conserved residues while the bacterial enzyme (α_2_β_2_) uses five different conserved residues that creates a much less polar environment than its dimeric counterpart (Valencia-Sánchez et al. 2016). The case of GlyRS presents a slightly different scenario than the example of LysRS covered above, as both forms descend from the ancestral class II synthetase enzyme. The simple hypothesis that both GlyRS forms arose from a common pre-GlyRS is highly unlikely, due to the aforementioned differences in the amino acid recognition residues, as well as other differences in motif 2 of the bacterial tetrameric enzyme that are not shared with any other of the other class II enzymes, except AlaRS. The AlaRS catalytic core presents the same differences as the tetrameric GlyRS (namely a highly conserved Glu residue in motif 2 is changed to Asp in AlaRS and GlyRS and a conserved Trp is involved in amino acid recognition), and their active sites share similar overall architectures. This observation led to the proposal that the dimeric form evolved from the ancestral class II enzyme while the tetrameric GlyRS evolved from either AlaRS or an ancestor of AlaRS that was able to aminoacylate both Ala and Gly. Due to this intimate evolutionary relationship and the shared similarities, some authors have proposed tetrameric GlyRS and AlaRS to be grouped in a different subclass, IId (Valencia-Sánchez et al. 2016).

### Expanding the set of 20 aaRSs

#### Selenocysteine

More than 140 different amino acids have been identified in naturally occurring proteins, although outside of the 20 proteinogenic ones nearly all of them are the result of post-translation modifications (Uy and Wold 1977; Macek et al. 2019). There are only two known exceptions that are specifically decoded during protein synthesis, the noncanonical selenocysteine and pyrrolysine. Selenocysteine was the first noncanonical amino acid discovered outside the original 20 amino acids of the genetic code (Cone et al. 1976; Hatfield et al. 1982). Structurally, it is similar to cysteine except that the thiol group is replaced by a selenol group. Selenocysteine is often found at the active site of proteins involved in redox reactions, where the lower redox potential of the selenium compared to sulfur proves to be beneficial (Johansson et al. 2004; Reich and Hondal 2016). No SecRS enzyme has been identified to date and an indirect charging mechanism, similar to that of AsnRS and GlnRS mentioned above, is used instead, where selenocysteine is formed from serine already charged on the tRNA^Sec^ (Lee et al. 1989). This process is carried out by selenocysteine synthase (SelA) in bacteria (Leinfelder et al. 1988; Forchhammer et al. 1991) and *o*-phosphoseryl-tRNA kinase (PSTK) followed by Sep-tRNA:Sec-tRNA synthase in archaea and eukarya (Fig. 2C; Carlson et al. 2004; Kaiser et al. 2005; Yuan et al. 2006). Another atypical aspect of selenocysteine incorporation is the absence of an assigned sense codon in the genetic code. For selenocysteine, incorporation occurs at UGA stop codons (Chambers et al. 1986; Lee et al. 1989) identified by a nearby *cis* element termed SECIS (for selenocysteine insertion sequence) (Liu et al. 1998), a stem–loop structure in the mRNA (bacteria) (Zinoni et al. 1990; Heider et al. 1992; Chen et al. 1993) or a structure in the 3′ untranslated region, far removed from the UGA codon, in archaea and eukaryotes (Zinoni et al. 1990; Berry et al. 1991, 1993). Sec-tRNA^Sec^ is then delivered to the ribosome by specialized elongation factors SelB in bacteria (Rasubala et al. 2005; Yoshizawa et al. 2005) and eEFSec alongside protein cofactors in archaea and eukaryotes (Berry et al. 1993; Fagegaltier 2000; Tujebajeva et al. 2000).

#### Pyrrolysine

Pyrrolysine (Pyl) is the other known addition to the standard code of 20 amino acids and the most recent, being identified in 2002 (Srinivasan et al. 2002; Soares et al. 2005). Pyrrolysine was first reported in some genera of methanogenic archaea from the *Methanosarcina* family, which produce Pyl-containing methyltransferases that allow the methylation of coenzyme M, the penultimate step in the formation of methane from methylamines (DiMarco et al. 1990). As in the case of selenocysteine, the special chemical properties of pyrrolysine are used in the methyltransferase active site, via a proposed methylammonium adduct that activates methylamines (Krzycki 2004). Unlike selenocysteine, pyrrolysine exists as a free metabolite and is biosynthesized by three enzymes (PylB, C and D) and it is charged by its unique synthetase, pyrrolysyl-tRNA synthetase, PylS, directly onto the tRNA^Pyl^ (Blight et al. 2004). Based upon the structure of its catalytic core, PylRS is classified as a Class II enzyme, although it possesses a unique mechanism of tRNA recognition. tRNA^Pyl^ itself has several unusual characteristics, such as shortened variable loop containing only three nucleotides instead of the more common five, an extended acceptor stem with six instead of five nucleotides or a reduced linkage between the acceptor and stem and the D-loop, consisting of only one base rather than the regular two found in other tRNAs (Ueda et al. 1992; Soma and Himeno 1998; Srinivasan et al. 2002). Upon binding tRNA, PylRS does not recognize the anticodon as an identity element but the adjacent bases instead. Similar to the case of selenocysteine, Pyl-tRNA^Pyl^ is cotranslationally inserted via specific amber UAG stop codons but unlike selenocysteine, Pyl-tRNA^Pyl^ is efficiently recognized by elogantion factor Tu and does not require any accessory factors (Krzycki 2004). It was also reported that the Pyl-containing methyltransferases from the *Methanosarcina* family contain a conserved sequence immediately 3′ of the UAG codon, predicted to form a stem–loop. It has been proposed that this sequence, termed the pyrrolysine incorporation sequence (PYLIS) (Namy et al. 2004; Théobald-Dietrich et al. 2005), would function as a contextual element for Pyl insertion, similar to the SECIS element for selenocysteine. It has also been reported that Pyl incorporation can occur in the absence of the PYLIS motif (Polycarpo et al. 2006; Longstaff et al. 2007), although it has been proposed that this effect is a consequence of codon supression, as Pyl-tRNA^Pyl^ can bind EF-Tu (Théobald-Dietrich et al. 2004). Since its discovery in methanogenic archaea, genes encoding PylRS have also been found in some bacteria, although in the latter the enzyme is coded by two genes *pylSn* (encoding for the N-terminal domain) and *pylSc* (encoding the carboxy-terminal domain). The restricted distribution of PylRS makes it difficult to ascertain the evolutionary history of the enzyme, although it has been proposed that PylRS arose in a pre-LUCA environment (Fournier et al. 2011) and later disseminated through horizontal gene transfer (Krzycki 2004; Fournier 2009; Fournier et al. 2011).

## TRANSLATION FIDELITY AND QUALITY CONTROL

### tRNA recognition

In order to ensure the faithful translation of the genetic message, synthetases must identify and pair particular tRNAs with their cognate amino acid which relies on the proper recognition of both substrates. This can prove extremely challenging for the synthetases as not only have they to discriminate the correct tRNA isoacceptor among a set of other tRNAs very similar in structure and chemical composition but also be able to select the cognate amino acid amidst an extremely large pool of similar amino acids, both proteinogenic and nonproteinogenic. The evolutionary pressure to maintain fidelity has driven aaRSs to develop an elevated specificity for their substrates, both the tRNA and the amino acid, although in some cases this specificity is tailored to particular environments of organisms or the properties of individual cellular compartments (Reynolds et al. 2010b; Yadavalli and Ibba 2012a). Through various structural and presteady state kinetic studies, a general model for tRNA binding has been elucidated (Beuning and Musier-Forsyth 1999). The first stage of tRNA binding is relatively fast and unspecific, driven mainly by the electrostatic interactions between positively charged residues of the proteins and the phosphate backbone of the tRNA (Tworowski et al. 2005; Perona and Hou 2007). Although the general cloverleaf structure is shared by all tRNAs, subtle differences in shape and conformation arise from sequence-dependent effects. These small differences in structure, charge distribution and base stacking allow for a first indirect readout of the phosphate and sugar backbone of tRNA by the synthetase, increase affinity of the enzyme for its cognate substrate and may provide a reason as to why the tRNA sequence is so heavily conserved, as even bases that do not directly interact with residues in the synthetase contribute nevertheless to this indirect readout (Perona and Hou 2007). After this initial phase, more specific contacts are made and the tRNA undergoes a slower conformational change during accommodation within the catalytic site. Differential binding affinity is not sufficient to ensure the correct recognition of the cognate tRNA and therefore kinetic discrimination is used to overcome these limitations and help the aaRS distinguish between cognate and noncognate tRNAs. Aminoacylation of the correct tRNA is influenced more by *k*_cat_ effects than by *K*_M_ effects (Ebel et al. 1973).

Proper recognition of the cognate tRNA issoaceptor is aided by identity elements which are certain nucleotides (in some instances modified) or structural elements, although their nature is idiosyncratic to each pair of synthetase and tRNA. These elements are called identity determinants if they promote the binding of the cognate tRNA or anti-determinants if they induce release of the noncognate tRNA and are usually located in the anticodon stem or in the acceptor arm (Kim and Quigley 1979; Normanly et al. 1992; Giege et al. 1998; Larkin et al. 2002; Pereira et al. 2018; Jordan Ontiveros et al. 2019). One major recognition element are bases 35, 36, and 37 of the anticodon stem–loop, usually heavily modified (Muramatsu et al. 1988; Perret et al. 1990; Pütz et al. 1994), as well as base 73 in the acceptor stem (Crothers et al. 1972; Giege et al. 1998; Paris et al. 2012). Because some amino acids are decoded by as many as six codons, some tRNAs that encode for the same amino acid may not share any nucleotide of the anticodon, which renders recognition difficult for the synthetase. Two examples are LeuRS and SerRS that have evolved alternative recognition mechanics to circumvent this issue. In the case of SerRS, the long variable arm of tRNA^Ser^ functions as an important discrimination element (Park and Schimmel 1988; Dock-Bregeon et al. 1990b; Asahara et al. 1993). One of the best studied examples of tRNA recognition is tRNA^Ala^, where recognition is exclusively based on a G3-U70 base pair (Hou and Schimmel 1988; McClain and Foss 1988). This base pair marks tRNA to be charged by AlaRS and is highly conserved, with the exact recognition mechanism varying between the three domains of life (Chong et al. 2018). This determinant is so robust that artificially transplanting this pair into other tRNAs triggers aminoacylation by AlaRS, both in vitro and in vivo (Hou and Schimmel 1988; McClain and Foss 1988; Francklyn and Schimmel 1989).

### Amino acid recognition

Recognition of the cognate amino acid poses a different challenge to tRNA, as amino acids are small molecules, often with similar physicochemical properties, making discrimination harder due to the limited number of contacts made between the substrate and the enzyme. To maintain accurate decoding, synthetases use a variety of strategies to discriminate against noncognate amino acids, such as exclusion via size, charge or use of metal ions that bind specific chemical groups. The PheRS synthetic active site harbors a conserved Ala residue that helps determine specificity for phenylalanine over tyrosine, although mitochondrial human and yeast PheRS lack this critical residue and rely on a higher specificity for cognate substrate to discriminate against the noncognate (Reynolds et al. 2010b; Bullwinkle et al. 2014a). Another well-studied example is recognition of glycine, the smallest amino acid which also lacks a side chain. The GlyRS active site is a highly negatively charged pocket, while one serine residue (eukaryotic) or two threonine residues (bacterial) prevent activation of any other amino acids larger than glycine (Valencia-Sánchez et al. 2016). Some aaRSs use coordinated metal ions in the active site to discriminate the cognate amino acid. Crystallographic studies in *E. coli* ThrRS have revealed the presence of a zinc atom within the active site, which plays an essential role in discriminating against the noncognate valine. The zinc atom is complexed with two residues of histidine, one residue of cysteine and a molecule of water into a tetrahedral coordination (Sankaranarayanan et al. 1999). Upon binding of threonine, the water molecule is displaced, and the zinc coordination changes from the tetrahedral to a square-based pyramidal pentacoordination state, stabilized by the amino and hydroxyl groups of threonine. The methyl group present in valine is unable to trigger this coordination change and is hence not activated by ThrRS (Sankaranarayanan et al. 2000). A similar mechanism is used by CysRS to discriminate against serine, in which a zinc atom within the active site establishes a tight thiol coordination with the cognate serine. This zinc-based recognition mechanism is so stringent that, as opposed to ThrRS, an editing activity is not required for maintaining accuracy in charging tRNA^Cys^ (Zhang et al. 2003). Similarly, the SerRS of methanogenic archaea possess a zinc ion tetra-coordinated by Cys and Glu residues and a molecule of water, which is displaced upon serine binding. Modeling threonine in the same orientation as serine onto the active site would induce clashes between the threonine methyl group and the Cys residues, forcing threonine into a less productive conformation for activation (Bilokapic et al. 2006, 2008).

### AaRSs and translational quality control

Faithful translation of the genetic message is paramount for the accurate synthesis of proteins and to prevent the introduction of mutations often associated with loss of function and disease. Gene expression is a complex process involving several steps from DNA replication, to transcription and finally translation, which use different strategies to maintain accuracy. The robust prevention, correction and repair activities of DNA polymerase keeps errors in replication at a low frequency of 1 per 10^−8^, while the proof-reading mechanisms of RNA polymerase allow a transcription error rate of about 1 in 10^−5^. The misincorporation of amino acids into a polypeptide chain at the ribosome accounts for most missense errors during translation, with some studies reporting estimates as high as 10^3^–10^4^ per amino acid site (Loftfield and Vanderjagt 1972; Ibba et al. 1997a). At this error rate, 15% of average-length proteins will contain an amino acid mismatch, which may be further increased under stress conditions such as starvation, viral infection or oxidative stress (Gomes et al. 2007; Netzer et al. 2009; Lant et al. 2018). This relatively low accuracy during protein synthesis at the ribosome is the result of two different events: mismatching of the mRNA:tRNA duplex and mischarging of the tRNA with a near- or noncognate amino acid. Faithful translation of an mRNA relies on accurate pairing of the codon–anticodon duplex via Watson–Crick interactions at the decoding center, buried deep within the ribosome. The tight grip that the decoding center exerts over the mRNA-duplex influences the stabilization of uncommon tautomeric forms of the nucleotides matching the dimensions of canonical Watson–Crick pairs, allowing formation of G–U pairs and subsequent introduction of mismatches in the resulting polypeptide (Rozov et al. 2015) and translation speed influences the occurrence of codon–anticodon mispairing, with more errors appearing at sites where ribosome velocity is higher (Mordret et al. 2019). Elongation factors also contribute to accurate decoding by selectively binding cognate aminoacyl-tRNAs. The binding of the elongation factor is thermodynamically tuned to bind the correct pair of amino acid:tRNA, while the decreased affinity for the noncognate pair may lead to premature release, and spontaneous hydrolysis, of the mischarged tRNA (LaRiviere et al. 2001; Blanchard et al. 2004; Gromadski and Rodnina 2004; Loveland et al. 2017). Nevertheless, while elongation factors contribute to fidelity, accurate protein synthesis is highly reliant on the availability of aminoacyl-tRNAs harboring the cognate amino acid, placing aaRSs as key players in maintaining fidelity (Mordret et al. 2019). The proofreading mechanisms used by aaRSs to ensure cognate aa-tRNAs are provided for translation are collectively referred to as “editing,” and are described in more detail below.

### Editing mechanisms in aaRSs

**FIGURE 3.**
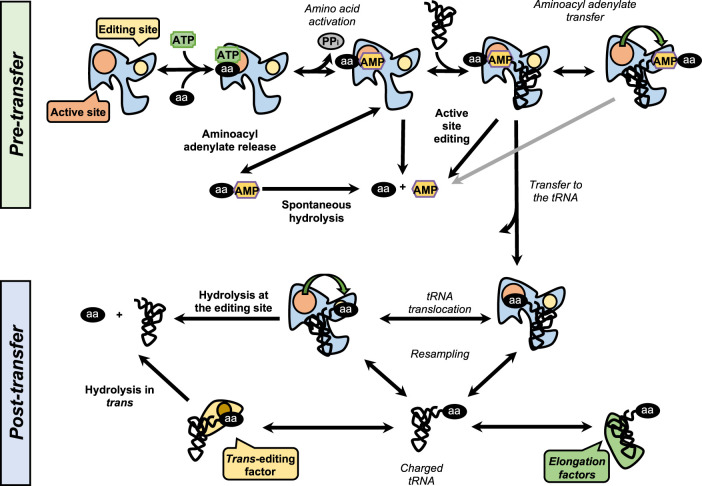
The editing pathways. Schematic overview of the editing pathways used by the synthetases. In the figure *above*, the events are in italics, while the editing paths are in bold. The pathways are divided between pretransfer and posttransfer pathways. In the pretransfer editing, the activated noncognate aminoacyl-adenylate may be released from the enzyme and hydrolyze spontaneously or be edited within the active site or a specialized active site. Upon transfer to the tRNA, the aminoacyl-tRNA can be translocated to the editing site or released and cleared by a dedicated *trans*-editing factor. The cognate aminoacyl-tRNA binds the elongation factors and proceeds to translation in the ribosome.

Despite the high affinity of aaRSs for their substrates, noncognate amino acids are sometimes activated and charged onto tRNAs, producing misacylated tRNAs that may, upon reaching the ribosome, be used in protein synthesis. In 1957, Linus Pauling estimated the misactivation rate about 1 in 200 from a theoretical standpoint (Pauling 1958), although later experiments by Loftfield (Loftfield and Vanderjagt 1972) showed the in vivo misincorporation rate to be closer to 1 in 3000. These results suggested the presence of some sort of proof-reading accounting for the difference before predicted and observed outcomes. These results led Fersht to propose a “double sieve” model in which the active site was a first sieve, able to exclude non cognate amino acid. Complementary to this was a second site, responsible for clearing the activated amino acid that surpassed the first filter, that possessed hydrolytic activity to clear the misacylated products that escape the first sieve (Fersht 1977). The existence of a separated editing site was described for the first time in class I IleRS (Baldwin and Berg 1966) and later in ValRS (Starzyk et al. 1987), where it is located in the CP1 domain and is responsible for clearing Val-tRNA^Ile^ and Thr-tRNA^Val^, respectively. To date, editing activity has been described in 10 out of the 23 aaRSs. In class I synthetases, this activity is located in the highly conserved CP1 domain, although in some enzymes such as MetRS the editing activity resides in the catalytic site (Nureki et al. 1998; Fukai et al. 2000). In class II synthetases, however, the editing domains are more idiosyncratic. Editing mechanisms can be divided in two categories: pre- or post-transfer editing, in regard to the editing taking place before or after the transfer of the amino acid to the tRNA (Fig. 3). Some aaRSs, such as ValRS and LeuRS, present both editing mechanisms (Nureki et al. 1998), but use one of them preferentially. Preferences on one of the two routes is also organism-dependent. *E. coli* LeuRS exclusively uses post-transfer editing while *S. cerevisiae* LeuRS predominantly uses the pretransfer activity (Mascarenhas et al. 2008). The use of either route is largely defined by the relative rates of aminoacyl adenylate hydrolysis and transfer to the tRNA. In systems such as ValRS, with a transfer rate around 200 times higher than hydrolysis, post-transfer editing is heavily favored (Fersht 1977; Dulic et al. 2010), whereas in enzymes where both rates are roughly equal, such as IleRS, both pathways contribute to clearing the noncognate product (Dulic et al. 2010; Minajigi and Francklyn 2010).

#### Pretransfer editing

Pretransfer editing has been described in both class I and class II aaRSs and takes place after aa-AMP synthesis but before the aminoacyl moiety is transferred to the tRNA. Although the tRNA does not participate in the reaction itself, it has been reported that tRNA binding promotes editing activity in some aaRSs and is a requirement in IleRS and LeuRS (Baldwin and Berg 1966; Boniecki et al. 2008; Yadavalli et al. 2008). Pretransfer editing can follow two main pathways. The first one is the selective release of the aa-AMP to the cytosol, where the labile phosphoesther bond is spontaneously hydrolyzed. The second route involves the enzymatic breakdown of the product and may happen either in the active site or in an independent editing site. Homocysteine, homoserine and ornithine are thiolated non proteinogenic amino acids that are routinely edited by several synthetases (MetRS, LysRS, and ValRS) via pretransfer editing activity located at the active site (Jakubowski 1991, 1994, 1999b). The architecture of the active site of MetRS partitions the substrates toward the aminoacylation or editing routes by establishing interaction with the side chain. In the case of the cognate methionine, the methyl side chain is stabilized with tryptophan and tyrosine residues and proceeds to aminoacylation. In contrast, homocysteine lacks this methyl side chain and interacts with an aspartate residue in the thiol subsite of the active site. This conformation triggers an intramolecular cyclization mechanism that produces homocysteine thiolactone, which is then expelled from the active site (Fersht and Dingwall 1979; Jakubowski 1991). Class II LysRS uses a similar mechanism for editing of homocysteine, homoserine and ornithine into homocysteine thiolactone, lactone and lactame, respectively (Jakubowski 1999a).

#### Post-transfer editing

Post-transfer editing takes place after the transfer of the amino acid to the tRNA and involves the hydrolysis of the ester bond, in a domain separated from the active site. The specific mechanism of editing is idiosyncratic to each synthetase but in general, once formed the aa-tRNA triggers a conformational change and the 3′ terminus containing the aa is translocated from the active site to the editing site, sometimes traversing distances as large as 40 Å (Silvian et al. 1999; Arnez et al. 2000; Fukai et al. 2000; Dock-Bregeon et al. 2004; Palencia et al. 2012). As the core of the tRNA remains bound to the enzyme, this translocation often involves a rearrangement of the 3′ terminus to relocate to the editing site. In class I synthetases, the CCA sequence of the tRNA adopts a hairpin conformation to enter the active site (Cusack 1997; Nureki et al. 1998), but must extend in order to reach the editing site while in class II synthetases, in a mirror fashion, the CCA shifts from an extended conformation at the synthetic site to a bent conformation to enter the editing site (Arnez et al. 2000; Dock-Bregeon et al. 2000). In class I synthetases with post-transfer editing activity, LeuRS, IleRS, and ValRS, this activity in confined to the CP1 domain while in class II synthetases these domains are more idiosyncratic. For example, AlaRS presents an editing domain at the carboxyl terminus while in bacterial ThrRS, a homologous domain is located in the amino terminus. This analogy between ThrRS and AlaRS illustrates once more that aaRSs are built by attaching different functional modules to the catalytic core (Sankaranarayanan et al. 1999). For ThrRS in archaea the amino-terminal editing domain is homologous to a family of enzymes called d-aminoacyl-tRNA-deacylases (DTDs) (Dock-Bregeon et al. 2000; Hussain et al. 2006), while in the heterotetrameric PheRS the editing domain is located in the β-subunit (Roy et al. 2004) and the catalytic activity is located in the α-subunit (Mosyak et al. 1995; Fishman et al. 2001). All these examples help in illustrating the diversity of mechanisms that have evolved to ensure accurate aminoacylation of tRNA. Several mechanisms by which the editing sites preferentially recognize the noncognate amino acid have been described. In some cases, the determining factor is size. The editing site of IleRS, for example, can accommodate a tRNA charged with the noncognate valine, but not if it is charged with the bigger Leu (Muramatsu et al. 1990). In fact, artificially enlarging the editing site allows leucyl-tRNA^Ile^ to be edited (Hendrickson et al. 2002). A similar mechanism has been established for the N2 editing site in ThrRS. Upon aminoacylation, the 3′ end of the tRNA harboring the amino acid undergoes a conformational change and moves from the active site to the N2 editing site, located 39 Å away from the catalytic site. The editing activity of ThrRS is directed toward eliminating the noncognate Ser-tRNA^Thr^, while editing of the cognate Thr must be prevented. The editing site architecture is a narrow pocket which is able to accommodate serine but not the bulkier threonine, as the methyl group clashes with the side chains of the amino acids at the editing site (Torres-Larios et al. 2002). In other cases, noncognate aa-tRNA editing is triggered by specific interactions with residues from the editing site. In PheRS, for example, the hydroxyl group present in the noncognate tyrosine interacts with residues of the editing site present in the β-subunit. As was mentioned before, release of the aa-tRNA is the limiting factor in class I synthetases, which make it possible for the tRNA to remain bound to the enzyme long enough for it to be edited. For class II synthetases, which rapidly release the product, these enzymes seem to be able to recapture the liberated misacylated tRNA. For example, PheRS is able to compete with EF-Tu and recapture and then edit Tyr-tRNA^Phe^ (Ling et al. 2007).

#### Editing factors

Another important component of the translation quality control machinery is the *trans*-editing family, freestanding proteins that are not synthetases but are in some cases homologs to the editing domains of such enzymes. The role of these *trans*-editing factors is to clear the misacylated tRNA before it reaches the ribosome, acting as additional checkpoints to ensure fidelity. In some archaeal genomes, the *thrS* gene is shortened and, as a result, the ThrRS encoded by it lacks the amino-terminal domain usually responsible for editing. This function is carried out by a freestanding protein termed ThrRS-ed, a structural homolog to the DTDs mentioned above (Beebe et al. 2004; Korencic et al. 2004; Dwivedi et al. 2005; Hussain et al. 2006; Shimizu et al. 2009). AlaX proteins can be found in the three kingdoms of life and are homologs to the editing domain of AlaRS (Chong et al. 2008). While AlaRS possesses editing activity against both Gly-tRNA^Ala^ and Ser-tRNA^Ala^, AlaX activity is exclusive to Ser-tRNA^Ala^. This apparent redundancy in activities highlights the enormous evolutionary pressure to prevent Ser misincorporation (Schimmel 2011). The INS superfamily are proteins homologs to the INS editing domain of ProRS with editing activity directed toward mischarged tRNA^Pro^. Examples of members of this family are the proteins ProXp-ala and Ybak which are responsible for editing Ala-tRNA^Pro^ and Cys-tRNA^Pro^, respectively. Although ProXp-ala (An and Musier-Forsyth 2004) is able to bind tRNA by interaction with the acceptor stem, Ybak requires binding to ProRS. Recently, the editing factors ProXp-ST1 and ProXp-ST2 were identified that edit tRNAs misacylated with Ser or Thr by several synthetases such as ThrRS, AlaRS, IleRS, LysRS, and ValRS (Liu et al. 2015; Chen et al. 2019).

Another family of *trans*-editing factors are the d-aminoacyl-tRNA-deacylases (DTDs), a set of *trans*-editing factors that specifically target tRNAs charged with d-forms of amino acids. Although the structure of the active site in synthetases can bind d-amino acids, it usually imposes a physical constraint that does not promote tRNA charging. In the cases in which D-amino acids are charged, their use in the ribosome must be prevented, as their introduction into the polypeptide chain may alter the three-dimensional structure of a protein. To date, two types of DTD are known with similar functions with subtle changes in sequences and possess a characteristic amino acid signature “SQFT” for DTD1 or “PQAT” for DTD2 (Wydau et al. 2009). In addition to editing d-amino acids, DTDs have also been shown to be able to hydrolyze the achiral glycine and edit Gly-tRNA^Ala^ (Pawar et al. 2017) while preventing editing of the cognate Gly-tRNA^Gly^ via a conserved discriminator base at position 73 (Kuncha et al. 2018).

### Substrate specificity and editing

Editing activity is not a requirement for all synthetases and, in fact, only about half of them possess this activity. In many instances, the high specificity of the active site is enough to circumvent the need for proofreading and editing. The overall need for accuracy seems to vary not only among organisms and can also differ between the analogous organellar and cytoplasmic enzymes. For example, in eukaryotes the cytosolic PheRS is a heterotetramer composed of 2 α subunits that harbor the active site and 2 β subunits with editing activity. The mitochondrial counterpart is only composed of the α subunit and lacks editing, which raises the question as to how protein synthesis accuracy is maintained in mitochondria. Although the mtPheRS is unable to clear the noncognate Tyr-tRNA^Phe^, it has been shown that the enzymes compensate with an enhanced specificity at the active site. The rate of misactivation of tyrosine is kept at a low rate of 1:7300 Phe:Tyr, compatible with an error rate in translation of 10^−4^ (Reynolds et al. 2010b). The need for this activity is dependent on the relative affinity for the cognate and noncognate amino acid. A very informative factor for evaluating the need for editing activity is the specificity coefficient, defined as the ratio of the catalytic efficiency (*k*_cat_/*K*_M_) for the cognate over the noncognate substrate. In aaRSs where this value is over 3000, the misincorporation of amino acid is assumed to occur at such a low level that it has no impact on cell fitness and editing activity is not needed (Fersht 1977; Mascarenhas et al. 2008; Reynolds et al. 2010a).

## AaRSs AND MISTRANSLATION

Although it is estimated that a cell is able to tolerate a miscoding event every 10^3^–10^4^ codons translated without compromising fitness (Yu et al. 2009), misincorporation of amino acids can lead to inactive or misfolded proteins, whose accumulation is associated with several pathologies. One such pathology is neurodegeneration, an abnormality that has been extensively studied in connection with mouse AlaRS. AlaRS is potentially able to facilitate misincorporation of Gly and Ser and prevents such errors with an editing domain at the carboxyl terminus that clears noncognate aa-tRNAs (Tsui and Fersht 1981; Beebe et al. 2003). In the mouse model, a missense mutation in this editing domain impairs editing activity and causes accumulation of mischarged tRNA^Ala^ in Purkinje cells, a population of neurons found in the cerebellum, leading to synthesis of misfolded proteins, triggering the activation of the unfolded protein response and, in turn, degeneration and cell death. Damage to the Purkinje cells in the mouse model provokes tremors, ataxia and impaired balanced (Lee et al. 2006).

AlaRS faces a unique challenge when maintaining fidelity as it is able to activate not only the smaller Gly but also the bigger Ser. Structural studies of AlaRS have shown that misactivation of serine is not a result of the activation pocket size but due to an interaction of the hydroxyl group of the serine with a conserved aspartate residue (Asp235 in *E. coli*) in the active site that is essential to hold the α-amino group of the cognate alanine (Guo et al. 2009). The particular architecture of the active site of AlaRS imposes an unavoidable constraint that makes activation of serine impossible to prevent. To avoid use of the noncognate Ser-tRNA^Ala^ in protein synthesis, an additional checkpoint has evolved, the aforementioned *trans*-editing factor AlaX/AlaXps, the activity of which is mainly focused against serine, and is also occasionally directed against Gly-tRNA^Ala^. Recently, an additional mechanism preventing Ser-to-Ala misincorporation has been described in the ANKRD16 protein. ANKRD16 is a vertebrate-specific, ankyrin repeat-containing protein that binds to the catalytic domain of AlaRS and is able to remove the misactivated serine in a tRNA-independent manner, incorporating the serine into ANKRD16 (Vo et al. 2018). The role that ANKRD16 plays in preventing serine mistranslation has not been described before for any synthetase, and although it may be tentatively classified as a *trans*-editing factor (or perhaps more accurately, cofactor) it represents a new layer of proofreading whose importance for limiting other synthetase aminoacylation is currently completely unknown.

While mistranslation is generally detrimental to viability, there are instances in which misincorporation of amino acids may actually provide a selective advantage under certain stress conditions (Pan 2013; Ribas de Pouplana et al. 2014). For example, MetRS has been shown to mismethionylate tRNAs in mammalian and yeast cells under oxidative stress conditions. Surface residues substituted with methionine could potentially neutralize the highly reactive species produced under oxidative stress, preventing the oxidation of sensitive amino acid chains at the active site that would result in permanent inactivation of the enzyme. The methionine-substituted residues would work as a sink for ROS without excessively compromising the folding of the protein (Netzer et al. 2009; Jones et al. 2011; Wiltrout et al. 2012). In *Mycobacteria*, errors in protein translation can promote the development of phenotypic resistance against the antibiotic rifampicin (Javid et al. 2014).

Maintaining and modulating accurate charging of tRNAs has important roles beyond its immediate impact on the fidelity of translation. The charging levels of tRNAs are used by cells to sense the available amino acid pool. Under amino acid starvation, uncharged tRNA accumulates in cells. In bacteria this deacylated tRNA enters the A site of the ribosome, the ribosome pauses as it is unable to proceed with translation and transfers the tRNA to RelA, which triggers production of (p)ppGpp, an alarmone that activates the stringent response in bacteria (Cashel 1969, 1975; Barker et al. 2001; Paul et al. 2004; Agirrezabala and Frank 2010; Arenz et al. 2016). During the stringent response, deep changes in translation, activation of transcription of amino acid synthesis genes and arrest of growth occurs, among other changes (Gallant et al. 1971; Rojas et al. 1984; Kanjee et al. 2011; Zhang et al. 2018). A deficient editing function can mask the real amino acid levels, as mischarged tRNAs do not activate the stringent response. For example, in *E. coli* mutants where the PheRS editing activity has been ablated, addition of the noncognate *m*-Tyr delays the onset of the stringent response. It has been proposed that this delay allows for additional rounds of cell growth and division, which may be advantageous to a subpopulation of cells (Bullwinkle et al. 2014b). A similar mechanism has been described in yeast, involving the general amino acid control (GAAC) pathway. Under amino acid starvation, deacylated tRNA interacts with the protein kinase general control nondepressible 2 (Gcn2p), activating a cascade that increases the expression of over 400 genes, decreasing translation and activating amino acid biosynthesis genes. Similar to bacteria, errors in the editing activity of PheRS lead to the accumulation of mischarged *m*-Tyr-tRNA^Phe^ and prevent accurate sensing of Phe starvation by the GAAC (Mohler et al. 2017).

Even though proofreading and editing are widely distributed in all three kingdoms of life, there are also examples where they have been lost during evolution. A recent study in *Microsporidia*, eukaryotic pathogens with the smallest known genome in eukaryotes, revealed synthetases that are shorter than their homologs from other eukaryotes. In several cases, these 40–300-aa-long deletions correspond to appended domains to the synthetases known to be involved in a diverse array of functions such as tRNA binding or catalytic efficiency or translation unrelated activities, such as the assembly of the MSC. One such deletion affects the editing domain of LeuRS, corrupting its proof-reading ability. As a result, misincorporation of Ile, Val, Met, the nonproteinogenic norvaline and several other amino acids were detected at Leu codons in proteomic analyses, accounting for up 5.9% of Leu codon missense translation (Melnikov et al. 2018a). Similarly, bacteria from the genus *Mycoplasma* have degenerate and inactive editing domains for ThrRS, PheRS and LeuRS and frequently exhibit noncognate amino acid incorporation in their proteomes (Li et al. 2011; Yadavalli and Ibba 2012b). Interestingly, although it is possible to find *Mycoplasma* genomes where two of these synthetase editing activities are missing no examples are known where three or more enzymes have lost their editing function, suggesting that there is an upper limit to tolerance for mistranslation. In the human fungal pathogen *Candida*, the identity of the CUG codon shifted from the canonical Leu to Ser. Because this tRNA has identity elements from both LeuRS and SerRS, the editing activity of LeuRS is unable to hydrolyze Leu-tRNA^Ser^. The ambiguous decoding can result in up to 5% of leucine misincorporation at Ser codons (Gomes et al. 2007; Silva et al. 2007).

The high level of tolerance to misincorporation exhibited by some organisms is the subject of intense studies. All the examples covered above are organisms with at least partially, parasitic lifestyles, which has led several authors to propose that misincorporation may increase phenotypic diversity, increasing the variability of protein synthesis. The resulting exponential expansion of the proteome could offer advantages against the immune system's defenses, which strongly rely on polypeptide presentation through the mayor histocompatibility complex (MHC) to detect pathogens (Miranda et al. 2013). This hypothesis is further strengthened upon extending the analysis to bacterial genomes, where phylogenetic analysis revealed that organisms with small genomes usually contain synthetases with mutated or degenerated editing sites. These bacteria possess highly reduced genomes and are either host-restricted or intracellular parasites (Melnikov et al. 2018b). The term statistical proteome has been coined to refer to those instances of organisms with such intrinsic variability although its impact on cell viability and pathogenesis remains to be elucidated (Gomes et al. 2007; Li et al. 2011).

## ROLES OF AMINOACYL-tRNA SYNTHETASES BEYOND TRANSLATION

In the decades after their initial discovery, the synthases were extensively characterized and their roles in tRNA charging analyzed in great detail. During that time, it has become clear that synthetases also exert a myriad of functions outside their classical role in tRNA charging, which is often referred to as “moonlighting.” Characterization of these functions outside translation is one of the fastest growing subfields of synthetase studies and, to date, there is evidence that synthetases participate in a plethora of different functions, ranging from gene regulators to biosynthetic activities, from intron splicing to angiogenesis (Guo et al. 2010; Pang et al. 2014; Mirande 2017; Yakobov et al. 2018). In hindsight, it may have been expected for these ancient enzymes to have diversified to exert so many functions, as the properties of their catalytic core, that is, the ability to bind nucleic acids in a highly specific mode and to efficiently discriminate chemical groups, along with their modular nature and the propensity to acquire extra domains make these enzymes an extremely versatile scaffold (Pang et al. 2014).

Perhaps the most straightforward examples of the functions of synthetases outside translation are those based on their ability to recognize the specific sequences of a tRNA. Participation of aaRSs in transcription or translation process is often achieved via hairpins and loops in the nucleic acid sequence that folds into cloverleaf-like structures that mimics those of the tRNA substrate. For example, *E. coli* ThrRS acts as a transcriptional regulator by repressing the translation of its own mRNA, which contains a region upstream of the Shine–Dalgarno sequence that forms two stems loops that mimic the tRNA^Thr^ anticodon arm and prompt ThrRS binding. These two stem loops contain both the anticodon CGU and two G and U conserved residues that act as identity elements for tRNA^Thr^ (Springer et al. 1988, 1989). Upon binding, the mRNA is captured by the enzyme, whose amino terminus clashes with the platform of the S30 subunit and prevents formation of the preinitiation complex while ThrRS is bound (Torres-Larios et al. 2002). Consistent with this model, deletion of the amino-terminal region abolishes the regulatory functions of ThrRS (Caillet et al. 2003). The competition for ThrRS between tRNA^Thr^ and the mimic leader sequence of the mRNA establish a regulatory feedback loop that matches ThrRS levels to the availability of tRNA^Thr^. These stem loops resemble tRNA^Thr^ so faithfully, that switching the anticodon mimicry from threonine to methionine is sufficient to change the operator to MetRS (Brunel et al. 1993). It has also been reported that in vertebrates, ThrRS has aquired a role in translation initiation, via its unique N-terminal extension that allows the enzyme to form a complex with 4EHP protein and recruit the initiation factor eIF4A (Jeong et al. 2019).

A similar model has been proposed for AspRS regulation in *S. cerevisiae*, in which the mRNA for AspRS contains in its upstream region two structured domains that display a tRNA^Asp^ anticodon-like stem–loop structure and a double-stranded helix (Ryckelynck et al. 2005b). In this model, two monomers of AspRS recognize and bind these tRNA-like structures, reducing the translation of mRNA and therefore regulating levels of AspRS (Ryckelynck et al. 2005a). As in the case of ThrRS, exploiting the ability of synthetases to bind tRNA-like structures provides a strategy successfully used by both prokaryotes and eukaryotes.

In fungi, TyrRS and LeuRS can participate in intron splicing by interacting with structural motifs similar to tRNAs (Mohr et al. 2001). In the fungi *Neurospora crassa*, the mitochondrial TyrRS has been shown to be involved in class I intron processing (Akins and Lambowitz 1987) via a specialized amino-terminal domain (Kittle et al. 1991). Although it was initially thought that the intron would adopt a tRNA-like folded structure, attempts to crystalize the complex have shown that the enzyme serves as a scaffold for the RNA, that binds both monomers of the synthetases across an RNA-binding surface different from that which binds tRNA^Tyr^ (Paukstelis et al. 2008). A similar splicing activity has also been described for the mtTyrRS of the Pezizomycotina fungi (Lamech et al. 2016) as well as in the yeast mitochondrial LeuRS (Houman et al. 2000).

In several instances, paralogs of synthetases take part in diverse activities, such as HisZ, a paralog of HisRS that is involved in the first step of histidine biosynthesis. YadB is a paralog of GluRS that attaches a glutamate to a queuosine modification (Salazar et al. 2003). Synthetases are also involved in regulation of nonsynthetases genes. One elegant model has been described in the yeast *S. cerevisieae*, in which the cytosolic MetRS and GluRS are found in a complex held together by the anchor protein Arc1p. Upon switching from fermentation to respiration, the Snf1/4 glucose sensing pathway is activated and expression of Arc1p is severely reduced, which prompts the release of free cytosolic MetRS and GluRS (Frechin et al. 2014). After being released, the cytosolic GluRS is imported to the mitochondria where it generates the noncanonical Glu-tRNA^Gln^, which is subsequently converted to Gln-tRNA^Gln^ by the mitochondrial amidotransferase, GatFAB, becoming the sole source of Gln-tRNA^Gln^ in the mitochondria (Frechin et al. 2009, 2010). At the same time, release of MetRS from the dissociated complex unmasks a nuclear localization signal and MetRS is therefore imported to the nucleus where it induces the expression of Atp1 subunit of the F1 domain of the ATP synthase, which is later exported into the mitochondria. The rest of the components are synthesized within the mitochondria, using the essential Gln-tRNA^Gln^ produced by the cytosolic GluRS. The concerted activities of the synthetases result in a coordinated expression of the components of the respiratory chain from two completely different organelles (Frechin et al. 2014). In eukaryotes, several regulatory functions have been described, but as almost half the synthases are forming part of the multisynthetase complex, those functions will be addressed briefly in the next section. All the examples provided above offer glimpses of the plethora of functions beyond translation that synthetases are often involved with and the reader is directed to addtional comprehensive reviews for further information (e.g., Pang et al. 2014).

## THE MULTISYNTHETASE COMPLEX

In bacteria, aaRSs are usually freestanding proteins, while in some Archaea associations between synthetases have been reported, usually comprising two or three synthetases working in a concerted manner. In the archaeon *Methanothermobacter thermoautothrophicus*, LysRS and ProRS anchor to the idiosyncratic amino- and carboxyl termini of LeuRS respectively, forming a synthesase complex alongside elongation factor 1A that enhances aminoacylation activity of both ProRS and LysRS (Praetorius-Ibba et al. 2007; Hausmann and Ibba 2008). A partnership between ArgRS and SerRS has also been reported in that same organism, where it enhances the catalytic activity of SerRS, especially under conditions of elevated temperature and osmolarity (Godinic-Mikulcic et al. 2011).

**FIGURE 4.**
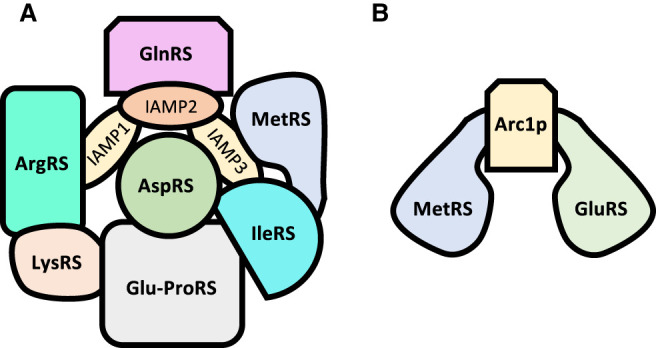
The multisynthetase complex. Schematic representation of the multisynthetase complex showing the differences of complexity between mammalian (*A*) and yeast (*B*). Mammalian MSC is a massive complex composed of nine synthetases and three accessory proteins, while the yeast counterpart is composed of two synthetases and a connecting protein. For the sake of simplicity, interactions are not shown; neither is the possible homodimerization of some of the components. The spatial arrangements and sizes of the components do not necessarily reflect their relative positions in the complexes.

In Eukarya, it is common for synthetases to assemble along with several accessory proteins into a macromolecular complex termed the multisynthetase complex (MSC) (Mirande et al. 1985; Kerjan et al. 1994; Han et al. 2003). The size and composition of the MSC vary depending on the organism. In the yeast *Saccharomyces cerevisiae*, the MSC is comprised of MetRS, GluRS and the auxiliary protein Arcp1 while in mammals there exists a large complex consisting of nine synthetases: Gln, Pro-Glu (in humans, ProRS, and GluRS are fused via a WHEP domain (Cerini et al. 1991), Ile, Leu, Met, Lys, Arg, and Asp and three nonenzyme components, the accessory interacting multifunctional proteins (AIMPs) 1-3 (Fig. 4). This striking difference in size and composition suggest an evolutionary link between the MSC expansion and the increased complexity of the interaction network of their components. Although the exact function of the MSC remains to be elucidated, it has been proposed that the association of different synthetases helps with the channeling of the amino acid substrate to the tRNA and further delivery to the ribosomal machinery (Kyriacou and Deutscher 2008). For example, it is known that AIMP3, which is specifically bound to MRS, relays the methionylated initiator tRNA to the initiator complex (Kang et al. 2012). One aspect that is fundamentally clear is that in addition to the canonical tRNA charging activity, the MSC components are involved in several fundamental processes outside of translation such as transcription, cell-signaling and tumorigenesis (Hyeon et al. 2019). For example, it has been shown that LeuRS interacts with the Target of Rapamycin (TOR) pathway, a highly conserved metabolic pathway involved in regulation of several key processes such as energy metabolism, protein synthesis, nutrient uptake or autophagy. In humans, LeuRS acts as an intracellular leucine sensor, acting as an activator of the mTORC1 complex (Bonfils et al. 2012; Durán and Hall 2012; Han et al. 2012).The overall architecture of the MSC has not yet been resolved, although some structures of subcomplexes have been crystalized (Norcum and Warrington 1998; Norcum 1999; Wolfe et al. 2005; Cho et al. 2015). The intricate architecture of the MSC is held together by a variety of domains appended to the synthetases as well as structural proteins. For instance, GST-homology domains are found inserted in Pro-GluRS, MetRS and AIMP 2 and 3 while WHEP domains are inserted in Pro-GluRS and MetRS. Leucine zipper motifs as well as helical motifs are found at the amino terminus of LysRS, ArgRS, and LeuRS as well as the AIMP proteins. All these motifs form a complex web of interactions that hold together all the elements. The auxiliary proteins AIMP 1–3 function as a scaffold to bind the synthetases and are fundamental for the assembly of the MSC and are essential for the complex to form (Shalak et al. 2001; Han et al. 2003; Mirande 2017). Deletion of AIMP2 triggers the disintegration of the complex and is associated with DNA damage and lethality (Han et al. 2008). Although it is crucial for the components to stay assembled, under certain conditions such as under stress, some of the members dissociate from the MSC and can perform other functions. AIMP3, for example, is usually bound to MetRS and serves as a conduit for the passage of the methionine-charged tRNA to initiation complex. Under DNA damage, however, GCN2 kinase phosphorylates MetRS, inducing a conformational change that forces the ejection of AIMP3, that is then mobilized to the nucleus and activates p53, activating DNA replication and repair processes. These auxiliary proteins are also involved in several diseases in ways that are still unclear, while AIMP2 exerts a potent antitumorigenesis activity through interaction with TGF-β, TNF-α, and p53 (Yum et al. 2016).

## AaRSs IN DISEASE

While the changes in the integrity of the MSC plays pivotal roles in several diseases, the synthetases have direct and wide-ranging central roles in an ever-increasing number of pathologies. One example is EPRS, which has been implicated, among other things, in adiposity, antiviral immunity and inflammation (Jia et al. 2008; Arif and Fox 2017). EPRS is phosphorylated at two serines located in the noncatalytic segment connecting both active sites upon activation by IFN-gamma (Arif et al. 2009). The phosphorylation causes EPRS to dissociate from the complex and bind three other proteins to form the cytosolic IFN-γ-activated inhibitor of translation (GAIT) complex, that silences translation of several genes involved in inflammatory processes (Arif et al. 2009).

Several synthetases other than EPRS, especially those in higher vertebrates, also have functions that extend beyond their essential role in translation, making the study of disease-associated mutations of aaRS quite a challenging endeavor. One of the best studied examples is Charcot–Marie–Tooth disease, a neurodegenerative disease in humans that is associated with a wide spectrum of neuropathies. Numerous genetic mutations are associated with CMT and, interestingly, these mutations are found in several aaRSs (for reviews, see Boczonadi et al. 2018; Wei et al. 2019), among which are the genes for GlyRS (Antonellis et al. 2003), TyrRS (Jordanova et al. 2006; Blocquel et al. 2017), LeuRS or AlaRS (Latour et al. 2010). An increasing body of results has also uncovered links with cancer, with almost half the synthetases connected directly or indirectly with cancer (for review, see Kim et al. 2013). For example, LysRS promotes anabolic cellular processes and growth regulating the mTORC1 pathway (Han et al. 2012), while ThrRS can be secreted by apoptotic cells and plays a role in angiogenesis (Arif et al. 2009). Other examples are the implication of LeuRS and MetRS in tumor formation or cell death induced by TrpRS (Shin et al. 2008; Lukk et al. 2010). In addition, other elements related to aaRSs, such as tRNA biogenesis, modification, elongation factors and ribosome biosynthesis are also implicated in several diseases (Tahmasebi et al. 2018) and synthetases have also been shown to play a role in MYC-driven growth (Zirin et al. 2019). Another potential connection between aaRSs and diseases comes with the recent finding that regulation of tRNA levels regulates the synthesis of tRNA-derived fragments (Torres et al. 2019). Another excellent example comes from the interplay between LysRS and the HIV-1 Gag polyprotein. During viral infection, HIV requires human tRNA^Lys^ as a primer for reserve transcription of genomic RNA to DNA, which is then packaged into the virus alongside human LysRS during viral assembly (Jiang et al. 1993; Cen et al. 2001; Musier-Forsyth 2019). Although several of the details are still unknown, it has been proposed that the Gag protein is able to capture newly synthetized LysRS before its assembly into the MSC (Guo et al. 2003; Halwani et al. 2004).

As mentioned above, eukaryotic organelles have their own sets of synthetases and tRNAs. Mutations in mitochondrial aaRSs disrupt the respiratory chain. These are the cause of multisystemic disorders such as encephalopathy, cardiomyopathy or sideroblastic anaemia, diseases that are fatal. While the main function of the mitochondria is to provide ATP, it is unclear how mutations in mitochondrial aaRSs can give rise to such a wide-ranging set of diseases, which include a large spectrum of clinical presentations, particularly muscular and neurological disorders, although the clinical manifestation seems to be related to the particular nature of the tissue. A considerable body of clinical and experimental data related to aaRS and tRNA-related mitochondrial myopathies has been generated over the last few years, and consistent themes are now starting to emerge although direct correlations between mutations, functional changes, and disease phenotypes remain elusive in many cases (González-Serrano et al. 2019; Wei et al. 2019). The roles of tRNAs and synthetases in health and disease will undoutebdly continue to expand, and are the subject of a series of recent thematic reviews (Musier-Forsyth 2019).

## DRUG DESIGN AGAINST aaRSs

Disruption of protein synthesis is an extremely widespread and effective strategy for development of antimicrobial drugs (Kohanski et al. 2010; Wilson 2014) and several of the most used antibiotics today specifically target ribosomal protein synthesis. Avoiding cross reaction of the antibiotic with the human protein synthesis machinery is a pivotal step in developing an effecting drug, a step which is eased while targeting the ribosome, as a result of the abundant structural differences between the 70S bacterial and 80S eukariotical ribosomes.

Due to their central role in protein synthesis, aminoacyl-tRNA synthetases have also garnered interest as potential antimicrobial targets. The abundance of structural and biochemical data for aaRSs has highlighted the evolutionary divergence between bacterial, archaeal and eukaryotic enzymes. The fact that aaRS presents three distinct sites for their substrates (plus the editing site in some cases) and that rely on very specific contacts for tRNA binding provide multiple avenues that can be exploited to abort tRNA charging. Comparison between sequences of aaRSs for the three domains of life revealed an early divergence in nine syntethases (PheRS, TyrRS, LeuRS, IleRS, GluRS, TrpRS, HisRS, ProRS, and AspRS) between bacterial and archaeal variants, with further divergence of the eukaryotic lineage, a phenomenon termed “full canonical pattern” (O'Donoghue and Luthey-Schulten 2003). Two particularly promising targets are GlyRS and PheRS, as the prokaryotic and eukaryotic variants exhibit prominent divergence, especially regarding quaternary structure. In addition, the fact that most synthetases in humans are locked into the MSC can make them less accessible to certain drugs.

Because the catalytic core of the aaRS is the most conserved sequence, design is often turned to the accessory sites such as the amino acid, ATP or tRNA binding pockets or the anticodon binding domain. One example of this is borrelidin, an experimental antimicrobial drug that inhibits ThrRS by interacting with all the amino acid, tRNA and adenylate binding sites, in adittion to a fourth binding site that is not required for substrate binding (Fang et al. 2015). Borrelidin has also been shown to exhibit antitumoral properties by inducing apoptosis of tumoral cells in leukemia (Habibi et al. 2012) as well as showing very promising results as an antimalarial drug (Azcárate et al. 2013).

Most of the inhibitors developed so far act as a noncleavable analog of the aminoacyl-tRNA. One of the best studied cases is muropicin, a broad-spectrum antibiotic that functions as an uncleaveable mimic of isoleucyl-adenilate and specifically inhibits eubacterial and archaeal synthetases (Hughes and Mellows 1980; Silvian et al. 1999). Interestingly, muropicin binds E. coli IleRS with over 8000-fold more strongly than rat IleRS despite only differing in two amino acids at the catalytic site (Nakama et al. 2001). The antiprotozoal halofuginone works in a similar fashion, mimicking both proline and tRNA^Pro^ in the active site (Zhou 2013) as is the case with phenyl-thiazolylurea-sulfonamides, that occupies the tRNA binding pocket of PheRS, inhibiting its activity (Abibi et al. 2014). On the other hand, agents such as pentamidine and purpuromycin have been described to inhibit different synthetases via nonspecific tRNA binding mechanisms (Kirillov et al. 1997; Sun and Zhang 2008). Another potential, yet largely unexplored, target is the editing site of the synthetases. As has been discussed above, some organisms (especially pathogens) seem to be able to withstand high levels of amino acid misincoporation, or even be able to dispense of the editing function altogether, making the editing a less promising target. Nevertheless, it has been described that the compound AN2690 (5-fluoro-1,3-dihydro-1-hydroxy-2,1-benzoxaborole, Tavaborole), a broad spectrum antifungal therapeutic agent, is able to inhibit LeuRS by trapping the tRNA within the editing site (Rock et al. 2007), forming a stable aduct that halts protein synthesis.

Drug design against aaRSs is steadily shifting from chemical library testing toward a more directed approach. As more and more structural and biochemical data are made available, specific interactions can be targets for drug design, allowing a more efficient and specific drug design. Within this framework, it is vital to identify tRNA determinants and residues involved in substrate binding, work that is made more difficult due to the still incomplete databases of tRNA modifications. To make matters worse, all compounds must be tested to avoid cross reactions not only with the cytosolic protein synthesis machinery, but with the mitochondrial one as well (Ho et al. 2018). Nevertheless, the ever growing list of publications regarding this topic validates aaRSs as an extremely interesting target for drug design.

## EXPANSION OF THE GENETIC CODE AND ARTIFICIAL AMINOACYL-tRNA SYNTHETASES

Beyond their natural roles, the aaRS have also played a pivotal role in synthetic biology, specifically the rewriting of the genetic code to allow for the incorporation of noncanonical amino acids (ncAAs, also known as nonstandard amino acids or nsAAs) into proteins. Incorporation of these engineered proteins into organisms allows an almost endless stream of possibilities: creation of biosensors and biomarkers, incorporation of new chemistries and new protein functions, advances in the study of protein structure and function, resistance to virus and horizontal gene transfer or containment of genetically modified organisms (Rovner et al. 2015). Successful expansion of the genetic code requires altering two fundamental steps in translation, repurposing an aaRS:tRNA pair to incorporate the ncAA and assigning a codon for this new translation component.

One of the biggest challenges when engineering an aaRSs to incorporate ncAAs is the issue of orthogonality, that is, creating an aaRS - amino acid pair that does not cross-react with other elements of the decoding machinery. One approach to solve this issue is the transplantation of an existing aaRS-tRNA pair from another organism. This is possible if the tRNA determinants for the donor and receiving species do not match. Exploiting the difference between tRNA identity elements from different species represented the first successful attempt to expand the genetic code with TyrRS. In bacteria, tRNA^Tyr^ identity elements include a G1-C72 base pair, which are inverted in archaea and eukaryotes (C1-G72) (Bedouelle 1990; Himeno et al. 1990; Fechter et al. 2000). Transplanting the tRNA^Tyr^/TyrRS from the archaea *Methanocaldococcus jannaschii* to *E. coli* allow the bacteria to encode an additional artificial amino acid O-methyl-L-tyrosine (Wang et al. 2001). Unfortunately, the introduction of this system in eukaryotes is not possible, as eukaryotic tRNA^Tyr^ recognition elements overlap with those of the archaeal tRNA^Tyr^ (Fechter et al. 2001).

By nature of their tRNA recognition elements, some aaRSs are inherently more suitable to become targets of genetic code expansion, as is the case of SerRS or LeuRS, whose tRNAs have important recognition elements situated in the variable arm rather than the anticodon, allowing the introduction of mutated codons without affecting tRNA charging (Park and Schimmel 1988; Dock-Bregeon et al. 1990a; Asahara et al. 1993). Such is the case of SerRS and tRNA^Sec^ in which the tRNA anticodon can be recoded without affecting aminoacylation, although in this case it requires construction of a tRNA^Sec^/tRNA^Ser^ hybrid, in which the elements for EF-Tu recognition from tRNA^Ser^ are transplanted into tRNA^Ser^, to prevent recognition by the specialized elongation factor SelB (Miller et al. 2015). The result is a chimeric tRNA that has been successfully used in developing the engineered tRNAs tRNA^UTu^ and tRNA^SecUX^. Another pair widely used is the PylRS-tRNA^Pyl^ system, as this amino acid is absent in most organisms and the distinctive structure of tRNA^Pyl^ and its idiosyncratic recognition elements allows for its introduction into a wide range of organisms (Kavran et al. 2007; Kobayashi et al. 2009; Ho et al. 2016). The tRNA^Pyl^–PylRS and *M. jannaschii* tRNA^Tyr^–TyrRS systems—either wild type or engineered—are so popular that more than two thirds of all the ncAAs incorporated to date are based on these systems (Melnikov and Söll 2019).

Achieving a robust orthogonality is just the first step in ncAA incorporation. The next step is the engineering of the synthetase to be able to activate the ncAA, which can be achieved by evolving the enzyme using mutagenesis strategies via either mutagenic oligos or phages, coupled with selection systems to sort out the candidates able to aminoacylate the ncAA. Examples of the first strategy include multiplex-automated genome engineering (MAGE) that is based on the incorporation of mutated oligos into the lagging strand during DNA replication (Wang et al. 2009; Isaacs et al. 2011). These oligonucleotides are designed to be complementary to specific regions of the desired gene (in the case of synthetases, these sites often include the amino acid or tRNA binding regions) and can be generated to include random sequences. Strategies based in phages, such as phage-assisted continuous evolution (PACE), use plasmid-based mutagenesis (Esvelt et al. 2011) to generate variability.

The final step is to assign a codon for the new ncAA tRNA/aaRS pair, a challenging task, as all triplet combinations have already been assigned an amino acid. As codon usage is not the same among all organisms (and, in fact, some organisms have stopped using certain codons altogether) it is possible to mutate the genome to reassign the least used codons to synonymous ones, freeing one triplet to be used for a ncAA. Another widespread strategy is the repurposing of stop codons, which has been achieved in *E. coli*, by replacing all TAG termination codons by TAA and removing release factor 1, providing a blank TAG codon that can be freely assigned (Isaacs et al. 2011; Lajoie et al. 2013). A similar approach has also been successfully used on other microbial genomes such as *Salmonella typhimurium* (Lau et al. 2017). This strategy can be expanded even further, recoding as much as seven codons without affecting cellular fitness significantly (Ostrov et al. 2016). Finally, it is also possible to add unnatural bases or codon quadruplets to make room in the genetic code for new tRNA-synthetase pairs (Anderson and Schultz 2003). With the advent of recent advances in whole genome synthesis, it is becoming possible to design and engineer whole genomes *de novo*, fine-tuned to incorporate all the necessities and constraints to build an efficient platform for ncAA incorporation. An example of this approach can be found in the design of the synthetic yeast genome Sc2.0, in which all TAG stop codons are changed to TAA and all tRNAs rearranged into a new chromosome (Mitchell et al. 2017; Richardson et al. 2017). The rapidly changing field of aaRS synthetic biology is well-served by a steady stream of comprehensive reviews for the interested reader covering a broad field of topics; from general overviews (Melnikov and Söll 2019; Smolskaya and Andreev 2019), genetic code expansion (Mukai et al. 2017b; Reynolds et al. 2017; Arranz-Gibert et al. 2018), adding amino acids with new chemistries (Wang et al. 2009; Liu and Schultz 2010; Sakamoto et al. 2014) or engineering of tRNA-aaRSs pairs (Mukai et al. 2017b; Baumann et al. 2018).

## CONCLUSION

Since their discovery in the 1960s, our knowledge about the aminoacyl-tRNA synthetases has expanded substantially. Research has provided information about the different strategies that have evolved over time to accurately perform aaRS function as proteomes grew in diversity and complexity, as well as provide meaningful insights on evolution from the LUCA to modern day organisms. Research on the impact of aaRSs on disease has implicated these enzymes in a plethora of diseases and provided new druggable targets and strategies to combat them. More recently, it has been shown that quality control and faithful translation have a broader impact beyond just protein synthesis and are involved in other biological processes. Quality control requirements vary within different organisms, which often must strike a balance between costs and adaptability, and some parasitic organisms are able to reduce their quality control to drastically increase their proteome variability. In recent years, the enzymatic versatility of the aaRSs has also gained considerable attention with the design of modified synthetases to incorporate artificial or nonnaturally occurring amino acids for synthetic biology applications. Overall, the aaRSs continue to impact more and more areas of molecular biology, belying the important but very limited role first assigned to them as the enzymes to charge Crick's adaptor molecules in his eponymous adaptor hypothesis.
